# Une surstructure de α-Ge, type diamant, induite par un dopage d’anti­moine

**DOI:** 10.1107/S2056989017004996

**Published:** 2017-04-04

**Authors:** Adrian Gómez Herrero, Lamia Hammoudi, Mohammed Kars, Thierry Roisnel, L. Carlos Otero-Diáz

**Affiliations:** a Centro de Microscopia Electrónica, Universidad Complutense, 28040 Madrid, Spain; b Université Houari-Boumedienne, Faculté de Chimie, Laboratoire Sciences des Matériaux, BP 32 El-Alia 16111 Bab-Ezzouar, Algeria; c Centre de Diffractométrie X, Sciences Chimiques de Rennes, UMR 6226 CNRS Université de Rennes 1, Campus de Beaulieu, Avenue du Général Leclerc, France; d Departomento Inorgánica, Facultad C.C. Químicas, Universidad Complutense, 28040 Madrid, Spain

**Keywords:** crystal structure, anti­mony, doped germanium, superstructure

## Abstract

Single crystals of anti­mony-doped germanium, Ge_1–*x*_Sb_*x*+0.01_ (*x* ≃ 0.0625), were grown by chemical transport reaction. The alloy crystallizes as a superstructure of diamond-type α-Ge. All atoms in the asymmetric unit lie on special positions and are characterized by strong covalent bonds. The anti­mony atoms substitute for one germanium atom at full occupancy at Wyckoff position 4*a* (site symmetry -43*m*), and are also at an adjacent tetra­hedral inter­stitial site with partially occupation (16%) at position 4*c* (or 4*d*) (site symmetry -43*m*). The structural model does not show close Sb⋯Sb contacts, and suggests that the inter­stitial anti­mony atoms move between the two adjacent tetra­hedral sites.

## Abstract 2

Single crystals of anti­mony-doped germanium were obtained by chemical transport reaction and characterized by X-ray diffraction. The structure crystallizes as a commensurate superstructure of the diamond-type α-Ge structure.

## Contexte chimique   

Le germanium a connu un regain d’attention ces dernières années comme matériau potentiellement promoteur d’améliorer des applications en micro et nanoélectronique (Claeys & Simoen, 2007 ▸). Ceci est du en grande partie à sa grande mobilité d’électrons et de trous. (Claeys *et al.*, 2010 ▸). En effet, le dopage du germanium peut affecter ses performances pour de futur applications électroniques, plus particulièrement l’anti­moine Sb, un dopant de type-*n* qui connait une attention particulière pour sa ultra-faible résistivité (Xu, *et al.*, 2016 ▸) et pour ces grandes performances n-MOSFET (Thareja *et al.*, 2010 ▸); d’où la nécessité de synthétiser des monocristaux Ge/Sb appropriés (Sheikhi *et al.*, 2016 ▸; Bruno *et al.*, 2010 ▸). L’étude du diagramme Ge-Sb montre la présence d’une seule phase métastable Ge_0,5_Sb_0,5_ tétragonale (Giessen & Gautier, 1972 ▸) et une solubilité Sb dans Ge qui varie entre 2.4–2.5 at.% (Okamoto, 2012 ▸).

## Commentaire structurelle   

**Figure 1:**
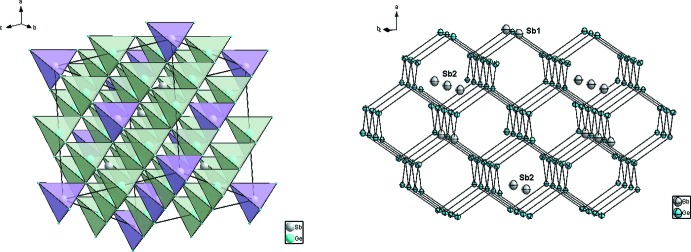
Structure de Ge_1–*x*_Sb_*x*+0,01_ (*x* ≃ 0,0625) montrant l’enchaînement des tètraèdres de coordination (*gauche*), et la position des atomes dopand Sb (droite). Pour plus de clareté, les ellipsoīdes de déplacement sont montrées à 95% de probabilité pour Ge et à 50% de probabilité pour Sb.

**Figure 2:**
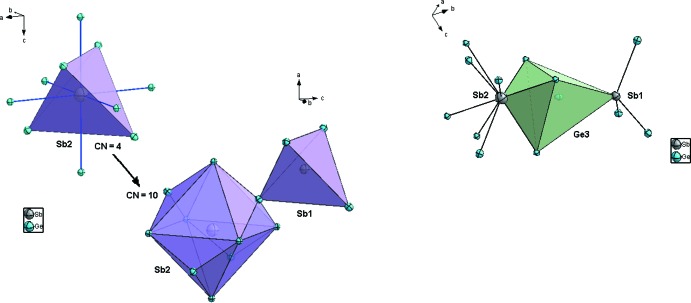
Polyèdres de coordination des atomes Sb2 (*gauche*) et Ge3 (droite) dans la structure de Ge_1–*x*_Sb_*x*+0,01_ (*x* ≃ 0,0625). Les ellipsoīdes de déplacement sont montrées à 95% de probabilité pour Ge et à 50% de probabilité pour Sb.

**Figure 3:**
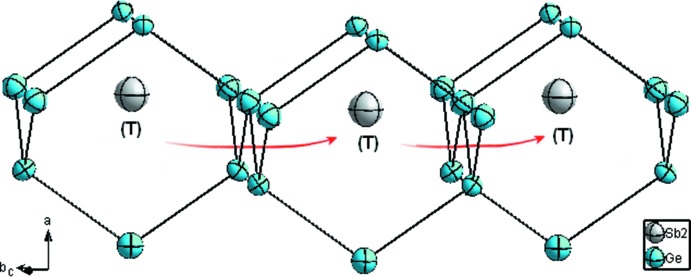
Mouvement des atomes Sb2 entre deux sites tétraédriques (T) adjacents. Les ellipsoīdes de déplacement sont montrées à 95% de probabilité pour Ge et à 50% de probabilité pour Sb.

L’alliage Ge_1–*x*_Sb_*x*+0,01_ (*x* ≃ 0,0625) cristallise selon une surstructure (2*a* × 2*a* × 2*a*) de type α-Ge diamant, *a* = 5,65675 (1) Å (Cooper, 1962 ▸), avec un volume de maille huit fois plus grand dans le groupe d’espace *F* 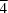
3*m*. Dans le système binaire Ge–Sb, les critères empiriques établis par Hume–Rothery pour la formation d’une solution solide ne sont pas complétements satisfaits (Rothery & Powell, 1935 ▸; Rothery, 1969 ▸; Rothery *et al.*, 1969 ▸), en particulier le premier critère qui spécifie que la différence de taille entre les deux atomes ne doit pas dépasser les 15% [*r*(Ge) = 1.23 Å et *r*(Sb) = 1,41 Å; Emsley, 1998 ▸]; ainsi la formation de surstructure est plutôt favorable (Rothery & Powell, 1935 ▸). Dans cette surstructure les atomes de Ge sont caractérisés, comme dans la structure de base, par de fortes liaisons covalentes et un environnement tétraédrique régulier (Fig. 1 ▸). Les distances Ge—Ge varient entre 2,437 (2)–2,444 (2) Å, et sont proches de celles observées dans la structure α-Ge type diamant (Emsley, 1998 ▸). L’atome Ge3 fait exception et posséde une coordinence plus élevée CN = 5 (bipyramide trigonale) par la présence de liaison Ge3—Sb2 (Fig. 2 ▸). Les études les plus récentes basées sur des méthodes dites *ab initio* montrent que lors du dopage du germanium par des atomes de type-*n* (tels que Sb, As et P), la position de substitution ainsi que la position tétraédrique d’insertion sont énergétiquement plus favorable (Maeta & Sueoka, 2014 ▸; Sluydts *et al.*, 2017 ▸), alors que la position de l’inter­stitiel dissocié (110) est plutôt favorable pour du germanium en auto-insertion (Moreira *et al.*, 2004 ▸). L’atome d’anti­moine Sb1 occupe la position 4*a* par substitution totale d’un atome de Ge, alors que l’atome Sb2 occupe partiellement la position tétraédrique (T) d’insertion 4*c* (ou 4*d*) (Fig. 1 ▸). Les atomes Sb dopants possèdent ainsi une coordinence tétraédrique avec de fortes liaisons covalentes comparables à celles des atomes Ge. Les distances Ge—Sb qui varient entre 2,435 (2)–2,448 (2) Å sont légèrement inférieures à la somme des rayons covalents 2,63 Å (Emsley, 1998 ▸), ce qui implique de fortes inter­actions. L’atome Sb2 se distingue par une deuxième sphère de coordination octa­édrique non distordue avec des distances Sb2—Ge2 de 2,8194 (9) Å, légèrement supérieures à la somme des rayons covalents, mais qui restent toutefois inférieures à la somme des rayons de van der Waals, *i.e.* 4,17 Å (Bondi, 1964 ▸), indiquant de faibles inter­actions. Cet atome Sb2 peut être ainsi caractérisé par une coordinance CN = 4 + 6 = 10 et des anti­prismes carrés à faces coiffés (Fig. 2 ▸). De courtes distances Sb—Sb ne sont pas observées, ce qui suggère que ces atomes préfèrent s’isoler dans le germanium sans former d’agglomérats. Cette tendance conforte le fait que les atomes Sb2 semblent mouvoir ou diffuser entre deux sites tétraédriques (T) adjacents (Fig. 3 ▸); phénomène déjà observé lors de l’étude par DFT du dopage du Ge par l’Al (Shi *et al.*, 2016 ▸), ou dans le cas du dopage du Si par les métaux de transitions (Matsukawa *et al.*, 2007 ▸). Enfin, comme observé dans la structure de base α-Ge diamant, on note un comportement isotrope de l’agitation thermique (ADP’s) des atomes, toute fois cette ADP’s est plus importante pour les atomes d’anti­moine.

## Enquête de base de données   

Dans le systeme Ge–Sb, un examen bibliographique montre l’existence d’une seule phase Ge_0.5_Sb_0.5_ (Giessen & Gautier 1972 ▸), dont la coordinence des atomes (CN = 4 + 2) peut être interprétée comme étant intermédiaire entre celle observée dans les structures de Sb (CN = 3 + 3) et de α-Ge (CN = 4 + 12). Cette recherche montre aussi l’existence de solutions solide dans α-Ge, comme c’est le cas dans les systemes Ge–Si (Dismukes *et al.*, 1964 ▸) ou dans Ge–Sn (Chizmeshya *et al.*, 2003 ▸), alors que dans le cas de Ge–Sb ce n’est réalisable que par mécanosynthèse; le cas de l’alliage Ge_34_Sb_66_ (Rebelo *et al.*, 2013 ▸).

## Synthèse et cristallisation   

**Figure 4:**
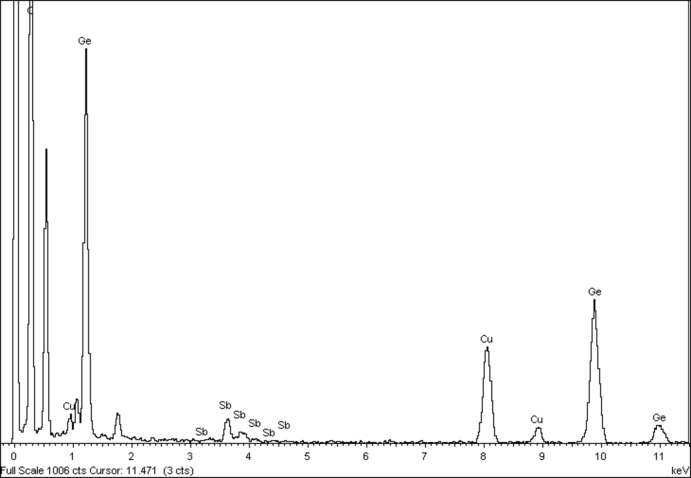
Spectre d’analyse par MET confirmant la présence des deux éléments chimiques attendus Ge et Sb.

Les monocristaux de l’alliage Ge_1–*x*_Sb_x+0,01_ (*x* ≃ 0,0625) ont été obtenus par la méthode de transport en phase vapeur lors des essais de synthèse du clathrate I_8_Sb_10_Ge_36_ (Kars *et al.*, 2010 ▸) à partir d’un mélange d’éléments purs et à une température de 1100 K pendant environ une semaine. L’anal­yse chimique par spectroscopie à rayons X à dispersion d’énergie (EDX Oxford Instruments) sur MET de plusieurs cristaux, confirme la présence des deux éléments Ge et Sb (Fig. 4 ▸).

## Affinement   

La structure a été résolue, dans le groupe d’espace *F* 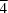
3*m* (sous-groupe de *Fd* 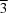
m de α-Ge diamant) grâce à l’algorithme *charge flipping* implanté dans le logiciel *SUPERFLIP* (Palatinus & Chapuis, 2007 ▸). Les détails de données cristallines, collection de données et affinement sont résumés dans le tableau 1 ▸. Une synthèse de Fourier-différence révèle la présence de deux pics d’intensité équivalente ρ_max_ = 8,34 et 8,17 e Å^−3^ situés à des distances inter­atomiques des atomes Ge3, ce qui a permis de localiser les atomes Sb2 dans deux sites tétraédriques 4*c* et 4*d*. Le meilleur résultat est obtenu avec des atomes Sb2 occupant ces deux sites séparément et conduisant à un faible taux d’occupation de 16%. Les atomes de Sb2 sont probablement distribués de manière statistique sur ces deux sites, mais les essais de paramétrer ce désordre sur ces deux sites ont échoués, ce qui explique en partie la carte de densité électronique obtenue en fin d’affinement: ρ_max_ = 3,59 e Å^−3^ (localisée à 2,45 Å de Ge3) et ρ_min_ = 1,54 e Å^−3^ (localisée à 1,11 Å de Sb2). La composition de l’alliage obtenue en fin d’affinement Ge (at%) = 92,81; Sb (at%) = 7,19] est proche de celle déduite par analyse chimique par MET [Ge (at%) = 92,02; Sb (at%) = 7,98] . L’affinement du paramétre de Flack (1983 ▸) suggère la prèsence d’une macle par inversion, la fraction en volume des composants est 0,56 (11):0,44 (11).

## Supplementary Material

Crystal structure: contains datablock(s) global, I. DOI: 10.1107/S2056989017004996/vn2127sup1.cif

Structure factors: contains datablock(s) I. DOI: 10.1107/S2056989017004996/vn2127Isup2.hkl

CCDC reference: 1541258

Additional supporting information:  crystallographic information; 3D view; checkCIF report

## Figures and Tables

**Table 1:** Détails expérimentaux

Données crystallines	
Formule chimique	Ge_0,9375_Sb_0,0725_
*M*_r_	4921,1
Système cristallin, groupe d’espace	Cubic, *F* 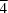 3*m*
Température (K)	293
*a* (Å)	11,276 (4)
*V* (Å^3^)	1433,6 (10)
*Z*	64
Type de rayonnement	Mo *K*α
μ (mm^−1^)	33,03
Taille des cristaux (mm)	0,48 × 0,37 × 0,16

Collection de données	
Diffractomètre	Bruker APEXII
Correction d’absorption	Multi-scan (*SADABS*; Sheldrick, 2002 ▸)
*T*_min_, *T*_max_	0,001, 0,005
Nombre de réflexions mesurées, indépendantes et observées [*I* > 3σ(*I*)] reflections	3308, 229, 123
*R*_int_	0,079
(sin θ/λ)_max_ (Å^−1^)	0,681

Affinement	
*R*[*F* > 3σ(*F*)], *wR*(*F*), *S*	0,058, 0,087, 1,79
Nombre de réflexions	229
Nombre de paramètres	16
Δρ_max_, Δρ_min_ (e Å^−3^)	3,59, −1,23
Absolute structure	Flack (1983 ▸), 99 paires de Friedel
Structure absolue Paramètre de structure absolue	0.44 (11)

Programmes informatiques:: *APEX2* (Bruker, 2006 ▸), *SUPERFLIP* (Palatinus & Chapuis, 2007 ▸), *JANA2000* (Petříček *et al.*, 2014 ▸) et *DIAMOND* (Brandenburg & Putz, 2009 ▸).

## supplementary crystallographic information

### Crystal data

**Table:**

Ge_0.9375_Sb_0.0725_	*D*_x_ = 5.698 Mg m^−^^3^
*M**_r_* = 4921.1	Mo *K*α radiation, λ = 0.71073 Å
Cubic, *F*43*m*	Cell parameters from 568 reflections
Hall symbol: F -4 2 3	θ = 3.1–26.7°
*a* = 11.276 (4) Å	µ = 33.03 mm^−^^1^
*V* = 1433.6 (10) Å^3^	*T* = 293 K
*Z* = 64	Prismatic, black
*F*(000) = 2157	0.48 × 0.37 × 0.16 mm

### Data collection

**Table:**

Bruker APEXII diffractometer	229 independent reflections
Radiation source: x-ray tube	123 reflections with *I* > 3σ(*I*)
Graphite monochromator	*R*_int_ = 0.079
CCD rotation images, thin slices scans	θ_max_ = 29.0°, θ_min_ = 3.1°
Absorption correction: multi-scan (SADABS; Sheldrick, 2002)	*h* = −14→15
*T*_min_ = 0.001, *T*_max_ = 0.005	*k* = −15→15
3308 measured reflections	*l* = −14→13

### Refinement

**Table:**

Refinement on *F*	(Δ/σ)_max_ = 0.0001
*R*[*F* > 3σ(*F*)] = 0.058	Δρ_max_ = 3.59 e Å^−^^3^
*wR*(*F*) = 0.087	Δρ_min_ = −1.23 e Å^−^^3^
*S* = 1.79	Extinction correction: B-C type 1 Gaussian isotropic (Becker & Coppens, 1974)
229 reflections	Extinction coefficient: 84 (8)
16 parameters	Absolute structure: 99 of Friedel pairs used in the refinement
Weighting scheme based on measured s.u.'s *w* = 1/(σ^2^(*F*) + 0.0001*F*^2^)	Absolute structure parameter: 0.44 (11)

### Special details

**Table:**

Refinement. The refinement was carried out against all reflections. The conventional *R*-factor is always based on *F*. The goodness of fit as well as the weighted *R*-factor are based on *F* and *F*^2^ for refinement carried out on *F* and *F*^2^, respectively. The threshold expression is used only for calculating *R*-factors *etc*. and it is not relevant to the choice of reflections for refinement. The crystal studied was twinned by inversion with a refined twin domain fraction of 0.56 (11):0.44 (11). The overlaps of reflection between the twin domains were calculated by Jana2000 software using the twinning matrix and user- defined treshold 0.1° for full overlap and 0.3° for full separation. Due to no support for twinning in the official CIF dictionary the twinning matrix has been saved in the CIF file using special _jana_cell_twin_matrix keyword. The program used for refinement, *Jana2000*, uses the weighting scheme based on the experimental expectations, see _refine_ls_weighting_details, that does not force *S* to be one. Therefore the values of *S* are usually larger than the ones from the *SHELX* program.

### Fractional atomic coordinates and isotropic or equivalent isotropic displacement parameters (Å^2^)

**Table:**

	*x*	*y*	*z*	*U*_iso_*/*U*_eq_	Occ. (<1)
Sb1	0	0	0	0.0141 (6)	
Ge1	0.12476 (19)	0.12476 (19)	0.37524 (19)	0.0032 (8)	
Ge2	0.25	0.25	0.49996 (8)	0.0026 (8)	
Ge3	−0.12467 (18)	−0.12467 (18)	−0.12467 (18)	0.0033 (8)	
Ge4	0	0	0.5	0.0039 (8)	
Sb2	−0.25	−0.25	−0.25	0.0141 (6)	0.162 (19)

### Atomic displacement parameters (Å^2^)

**Table:**

	*U*^11^	*U*^22^	*U*^33^	*U*^12^	*U*^13^	*U*^23^
Sb1	0.0141 (11)	0.0141 (11)	0.0141 (11)	0	0	0
Ge1	0.0032 (14)	0.0032 (14)	0.0032 (14)	−0.00013 (17)	0.00013 (17)	0.00013 (17)
Ge2	0.0027 (14)	0.0027 (14)	0.0025 (15)	0.0000 (2)	0	0
Ge3	0.0033 (14)	0.0033 (14)	0.0033 (14)	0.00019 (18)	0.00019 (18)	0.00019 (18)
Ge4	0.0039 (14)	0.0039 (14)	0.0039 (14)	0	0	0
Sb2	0.0141 (11)	0.0141 (11)	0.0141 (11)	0	0	0

### Geometric parameters (Å, º)

**Table:**

Sb1—Ge3	2.435 (2)	Ge1—Ge2^v^	2.443 (2)
Sb1—Ge3^i^	2.435 (2)	Ge1—Ge4	2.437 (2)
Sb1—Ge3^ii^	2.435 (2)	Ge2—Ge3^vi^	2.444 (2)
Sb1—Ge3^iii^	2.435 (2)	Ge2—Ge3^vii^	2.444 (2)
Ge1—Ge2	2.443 (2)	Ge2—Sb2^viii^	2.8194 (9)
Ge1—Ge2^iv^	2.443 (2)	Ge3—Sb2	2.448 (2)
			
Ge3—Sb1—Ge3^i^	109.47 (7)	Ge2^xv^—Sb2—Ge2^xvii^	90
Ge3—Sb1—Ge3^ii^	109.47 (7)	Ge2^xv^—Sb2—Ge3	125.26 (5)
Ge3—Sb1—Ge3^iii^	109.47 (7)	Ge2^xv^—Sb2—Ge3^xviii^	54.74 (5)
Ge3^i^—Sb1—Ge3	109.47 (7)	Ge2^xv^—Sb2—Ge3^xix^	125.26 (5)
Ge3^i^—Sb1—Ge3^ii^	109.47 (7)	Ge2^xv^—Sb2—Ge3^xx^	54.74 (5)
Ge3^i^—Sb1—Ge3^iii^	109.47 (7)	Ge2^x^—Sb2—Ge2^xv^	180
Ge3^ii^—Sb1—Ge3	109.47 (7)	Ge2^x^—Sb2—Ge2^xvi^	90
Ge3^ii^—Sb1—Ge3^i^	109.47 (7)	Ge2^x^—Sb2—Ge2^xi^	90
Ge3^ii^—Sb1—Ge3^iii^	109.47 (7)	Ge2^x^—Sb2—Ge2^xii^	90
Ge3^iii^—Sb1—Ge3	109.47 (7)	Ge2^x^—Sb2—Ge2^xvii^	90
Ge3^iii^—Sb1—Ge3^i^	109.47 (7)	Ge2^x^—Sb2—Ge3	54.74 (5)
Ge3^iii^—Sb1—Ge3^ii^	109.47 (7)	Ge2^x^—Sb2—Ge3^xviii^	125.26 (5)
Ge2—Ge1—Ge2^iv^	109.36 (8)	Ge2^x^—Sb2—Ge3^xix^	54.74 (5)
Ge2—Ge1—Ge2^v^	109.36 (8)	Ge2^x^—Sb2—Ge3^xx^	125.26 (5)
Ge2—Ge1—Ge4	109.59 (8)	Ge2^xvi^—Sb2—Ge2^xv^	90
Ge2^iv^—Ge1—Ge2	109.36 (8)	Ge2^xvi^—Sb2—Ge2^x^	90
Ge2^iv^—Ge1—Ge2^v^	109.36 (8)	Ge2^xvi^—Sb2—Ge2^xi^	90
Ge2^iv^—Ge1—Ge4	109.59 (8)	Ge2^xvi^—Sb2—Ge2^xii^	180
Ge2^v^—Ge1—Ge2	109.36 (8)	Ge2^xvi^—Sb2—Ge2^xvii^	90
Ge2^v^—Ge1—Ge2^iv^	109.36 (8)	Ge2^xvi^—Sb2—Ge3	125.26 (5)
Ge2^v^—Ge1—Ge4	109.59 (8)	Ge2^xvi^—Sb2—Ge3^xviii^	125.26 (5)
Ge1—Ge2—Ge1^ix^	109.70 (8)	Ge2^xvi^—Sb2—Ge3^xix^	54.74 (5)
Ge1—Ge2—Ge3^vi^	109.35 (7)	Ge2^xvi^—Sb2—Ge3^xx^	54.74 (5)
Ge1—Ge2—Ge3^vii^	109.35 (7)	Ge2^xi^—Sb2—Ge2^xv^	90
Ge1—Ge2—Sb2^viii^	125.15 (5)	Ge2^xi^—Sb2—Ge2^x^	90
Ge1^ix^—Ge2—Ge1	109.70 (8)	Ge2^xi^—Sb2—Ge2^xvi^	90
Ge1^ix^—Ge2—Ge3^vi^	109.35 (7)	Ge2^xi^—Sb2—Ge2^xii^	90
Ge1^ix^—Ge2—Ge3^vii^	109.35 (7)	Ge2^xi^—Sb2—Ge2^xvii^	180
Ge1^ix^—Ge2—Sb2^viii^	125.15 (5)	Ge2^xi^—Sb2—Ge3	54.74 (5)
Ge3^vi^—Ge2—Ge3^vii^	109.74 (8)	Ge2^xi^—Sb2—Ge3^xviii^	125.26 (5)
Ge3^vi^—Ge2—Sb2^viii^	54.87 (5)	Ge2^xi^—Sb2—Ge3^xix^	125.26 (5)
Ge3^vii^—Ge2—Ge3^vi^	109.74 (8)	Ge2^xi^—Sb2—Ge3^xx^	54.74 (5)
Ge3^vii^—Ge2—Sb2^viii^	54.87 (5)	Ge2^xii^—Sb2—Ge2^xv^	90
Sb1—Ge3—Ge2^x^	109.61 (8)	Ge2^xii^—Sb2—Ge2^x^	90
Sb1—Ge3—Ge2^xi^	109.61 (8)	Ge2^xii^—Sb2—Ge2^xvi^	180
Sb1—Ge3—Ge2^xii^	109.61 (8)	Ge2^xii^—Sb2—Ge2^xi^	90
Sb1—Ge3—Sb2	180	Ge2^xii^—Sb2—Ge2^xvii^	90
Ge2^x^—Ge3—Ge2^xi^	109.34 (8)	Ge2^xii^—Sb2—Ge3	54.74 (5)
Ge2^x^—Ge3—Ge2^xii^	109.34 (8)	Ge2^xii^—Sb2—Ge3^xviii^	54.74 (5)
Ge2^x^—Ge3—Sb2	70.39 (6)	Ge2^xii^—Sb2—Ge3^xix^	125.26 (5)
Ge2^xi^—Ge3—Ge2^x^	109.34 (8)	Ge2^xii^—Sb2—Ge3^xx^	125.26 (5)
Ge2^xi^—Ge3—Ge2^xii^	109.34 (8)	Ge2^xvii^—Sb2—Ge2^xv^	90
Ge2^xi^—Ge3—Sb2	70.39 (6)	Ge2^xvii^—Sb2—Ge2^x^	90
Ge2^xii^—Ge3—Ge2^x^	109.34 (8)	Ge2^xvii^—Sb2—Ge2^xvi^	90
Ge2^xii^—Ge3—Ge2^xi^	109.34 (8)	Ge2^xvii^—Sb2—Ge2^xi^	180
Ge2^xii^—Ge3—Sb2	70.39 (6)	Ge2^xvii^—Sb2—Ge2^xii^	90
Ge1—Ge4—Ge1^xiii^	109.47 (7)	Ge2^xvii^—Sb2—Ge3	125.26 (5)
Ge1—Ge4—Ge1^xiv^	109.47 (7)	Ge2^xvii^—Sb2—Ge3^xviii^	54.74 (5)
Ge1—Ge4—Ge1^ii^	109.47 (7)	Ge2^xvii^—Sb2—Ge3^xix^	54.74 (5)
Ge1^xiii^—Ge4—Ge1	109.47 (7)	Ge2^xvii^—Sb2—Ge3^xx^	125.26 (5)
Ge1^xiii^—Ge4—Ge1^xiv^	109.47 (7)	Ge3—Sb2—Ge3^xviii^	109.47 (7)
Ge1^xiii^—Ge4—Ge1^ii^	109.47 (7)	Ge3—Sb2—Ge3^xix^	109.47 (7)
Ge1^xiv^—Ge4—Ge1	109.47 (7)	Ge3—Sb2—Ge3^xx^	109.47 (7)
Ge1^xiv^—Ge4—Ge1^xiii^	109.47 (7)	Ge3^xviii^—Sb2—Ge3	109.47 (7)
Ge1^xiv^—Ge4—Ge1^ii^	109.47 (7)	Ge3^xviii^—Sb2—Ge3^xix^	109.47 (7)
Ge1^ii^—Ge4—Ge1	109.47 (7)	Ge3^xviii^—Sb2—Ge3^xx^	109.47 (7)
Ge1^ii^—Ge4—Ge1^xiii^	109.47 (7)	Ge3^xix^—Sb2—Ge3	109.47 (7)
Ge1^ii^—Ge4—Ge1^xiv^	109.47 (7)	Ge3^xix^—Sb2—Ge3^xviii^	109.47 (7)
Ge2^xv^—Sb2—Ge2^x^	180	Ge3^xix^—Sb2—Ge3^xx^	109.47 (7)
Ge2^xv^—Sb2—Ge2^xvi^	90	Ge3^xx^—Sb2—Ge3	109.47 (7)
Ge2^xv^—Sb2—Ge2^xi^	90	Ge3^xx^—Sb2—Ge3^xviii^	109.47 (7)
Ge2^xv^—Sb2—Ge2^xii^	90	Ge3^xx^—Sb2—Ge3^xix^	109.47 (7)

Symmetry codes: (i) *y*, −*x*, −*z*; (ii) −*x*, −*y*, *z*; (iii) −*y*, *x*, −*z*; (iv) *x*, −*z*+1/2, −*y*+1/2; (v) −*z*+1/2, −*x*+1/2, *y*; (vi) *y*+1/2, −*x*, −*z*+1/2; (vii) −*y*, *x*+1/2, −*z*+1/2; (viii) *x*+1/2, *y*+1/2, *z*+1; (ix) −*x*+1/2, −*y*+1/2, *z*; (x) *y*−1/2, −*x*, −*z*+1/2; (xi) *x*−1/2, −*z*+1/2, −*y*; (xii) −*z*+1/2, −*x*, *y*−1/2; (xiii) *y*, −*x*, −*z*+1; (xiv) *z*−1/2, *x*, *y*+1/2; (xv) *x*−1/2, *y*−1/2, *z*−1; (xvi) *z*−1, *x*−1/2, *y*−1/2; (xvii) −*x*, *z*−1, −*y*; (xviii) *y*, −*x*−1/2, −*z*−1/2; (xix) −*x*−1/2, −*y*−1/2, *z*; (xx) −*y*−1/2, *x*, −*z*−1/2.
